# Using Technology to Support Self-Managing Hypertension in African Americans

**DOI:** 10.1093/geroni/igaa057.1965

**Published:** 2020-12-16

**Authors:** Carolyn Still, Phuong Dang, Abdus Sattar

**Affiliations:** 1 Case Western Reserve University, Cleveland, Ohio, United States; 2 Case Western Reserve University, Frances Payne Bolton School of Nursing, Richmond Heights, Ohio, United States

## Abstract

The purpose of this study was to examine the effects of a community and technology-based intervention to support self-managing hypertension in African American (AA). Sixty AA with hypertension were randomly assigned to Coachman (a technology-based intervention) or Enhanced Usual Care. COACHMAN is comprised of blood pressure (BP) monitoring with study issued monitor, six-weeks of web-based education, training to use a medication management application, and nurse counseling. Data were collected on contextual factors (demographics, perceived social support), process factors (hypertension knowledge, self-efficacy, technology use/adoption), and proximal health behaviors (medication adherence, diet, exercise) at baseline, and 8 and 12 weeks. While mean difference in BP reduction was not statistically significant, we found that half of the subjects randomized to the intervention group had an average systolic BP reduction of 13.5 mmHg that we would regard as clinically significant. Interventions that incorporate mHealth can support self-managing hypertension in AA, and improve BP.

